# Muscle and Tendon Adaptation in Adolescence: Elite Volleyball Athletes Compared to Untrained Boys and Girls

**DOI:** 10.3389/fphys.2017.00417

**Published:** 2017-06-16

**Authors:** Falk Mersmann, Georgios Charcharis, Sebastian Bohm, Adamantios Arampatzis

**Affiliations:** ^1^Department of Training and Movement Sciences, Humboldt-Universität zu BerlinBerlin, Germany; ^2^Berlin School of Movement ScienceBerlin, Germany

**Keywords:** muscle, tendon, adaptation, athletes, adolescence, tendinopathy, imbalance

## Abstract

Though the plasticity of human tendons is well explored in adults, it is still unknown how superimposed mechanical loading by means of athletic training affects the properties of tendons during maturation. Due to the increased responsiveness of muscle to mechanical loading, adolescence is an important phase to investigate the effects of training on the mechanical properties of tendons. Hence, in the present study we compared vastus lateralis (VL) architecture, muscle strength of the knee extensor muscles and patellar tendon mechanical properties of male and female adolescent elite athletes to untrained boys and girls. Twenty-one adolescent volleyball athletes (A; 16.7 ± 1 years; 12 boys, 9 girls) and 24 similar-aged controls (C; 16.7 ± 1 years; 12 boys and girls, respectively) performed maximum isometric contractions on a dynamometer for the assessment of muscle strength and, by integrating ultrasound imaging, patellar tendon mechanical properties. Respective joint moments were calculated using an inverse dynamics approach and an electromyography-based estimation of antagonistic contribution. Additionally, the VL pennation angle, fascicle length and muscle-thickness were determined in the inactive state by means of ultrasound. Adolescent athletes produced significantly greater knee extension moments (normalized to body mass) compared to controls (A: 4.23 ± 0.80 Nm/kg, C: 3.57 ± 0.67 Nm/kg; *p* = 0.004), and showed greater VL thickness and pennation angle (+38% and +27%; *p* < 0.001). Tendon stiffness (normalized to rest length) was also significantly higher in athletes (A: 86.0 ± 27.1 kN/strain, C: 70.2 ± 18.8 kN/strain; *p* = 0.04), yet less pronounced compared to tendon force (A: 5785 ± 1146 N, C: 4335 ± 1015 N; *p* < 0.001), which resulted in higher levels of tendon strain during maximum contractions in athletes (A: 8.0 ± 1.9%, C: 6.4 ± 1.8%; *p* = 0.008). We conclude that athletic volleyball training provides a more efficient stimulus for muscle compared to tendon adaptation, which results in an increased demand placed upon the tendon by the working muscle in adolescent volleyball athletes. Besides implications for sport performance, these findings might have important consequences for the risk of tendon overuse injury.

## Introduction

Tendons transmit the forces generated by the muscle to the skeleton and, thus, feature a crucial role in the production of torques around joints for movement. The viscoelastic properties of tendons importantly contribute to the force- and power-generating capacity of the muscle-tendon unit by optimizing the operating range with regard to the force-velocity and force-length relationship and storage and release of mechanical strain energy (Hof et al., 1983; Ettema et al., 1990; Roberts, 1997; Kawakami and Fukunaga, 2006). Thus, there is a clear relationship between the properties of tendons and human movement performance, for example for running (Arampatzis et al., 2006; Fletcher et al., 2010; Albracht and Arampatzis, 2013), sprinting (Stafilidis and Arampatzis, 2007; Kubo et al., 2011b), jumping (Bojsen-Møller et al., 2005), rapid force production (Waugh et al., 2013) or balance recovery performance (Karamanidis et al., 2008).

On the other hand, the tissue deformation during loading makes the tendon susceptible for injury. Cyclic (or constant) high-magnitude strains applied to a tendon can cause fatigue damage and even rupture (Woo, 1982; Wren et al., 2003; Lavagnino et al., 2006; Legerlotz et al., 2013; Veres et al., 2013). Therefore, tendons are able to adapt to changes in their mechanical environment and, for example, respond to an increase in muscle force potential with a modulation of its mechanical properties. The increase of tendon stiffness in response to biologically effective repetitive mechanical stimulation over a certain time is mediated by changes of the material properties (Kubo et al., 2001; Arampatzis et al., 2007, 2010; Malliaras et al., 2013; Bohm et al., 2014) and, following long-term loading, radial tendon growth (Rosager et al., 2002; Magnusson and Kjaer, 2003; Kongsgaard et al., 2005; Couppé et al., 2013).

The plasticity of tendons is well explored in human adults (Bohm et al., 2015; Wiesinger et al., 2015). In contrast, there is basically no information on the adaptation of human tendons in response to mechanical loading during childhood and adolescence. Waugh et al. (2014) were the first to investigate the effects of mechanical loading on Achilles tendon adaptation in pre-pubertal children in a longitudinal study and found a significant increase of muscle strength and a concomitant increase of tendon stiffness following a resistance exercise intervention. However, puberty is associated with profound changes of the musculoskeletal and endocrine system, which affect the plasticity of muscle and likely tendon as well. Estrogen and particularly testosterone increase the anabolic responsiveness of muscle to mechanical loading (Vingren et al., 2010; Hansen and Kjaer, 2014) and a recent meta-analysis provided evidence for an increase of muscle strength plasticity during and after peak height velocity (Moran et al., 2016). Youth athletes feature markedly greater muscle size compared to untrained adolescents, as demonstrated by Kanehisa et al. (1995b, 2003) as well as Hoshikawa et al. (2011), and, though it has not been demonstrated thus far experimentally, it is likely that changes in muscle architecture also contribute to training-induced gains in strength in adolescents (Aagaard et al., 2001). How the change in the hormonal environment affects tendon adaptation is on the other hand virtually unknown and, thus, the findings of Waugh et al. (2014) on pre-pubertal children are not necessarily representative for pubertal children or adolescents. Second, the mechanical stimulus provided by Waugh and colleagues in their machine-based resistance training intervention (i.e., high magnitude loading, long contraction durations) is known to facilitate tendon mechanical properties (Bohm et al., 2015), yet the dominant type of loading for young athletes is sport-specific and might not automatically provide an efficient stimulus for both muscle and tendon adaptation.

As the incidence of tendon overuse injuries seems to increase during adolescence (Stracciolini et al., 2014; Simpson et al., 2016) and pose a major threat to athletes from jump disciplines (Lian et al., 2005), it is crucial (a) to deepen our understanding of the adaptive processes of muscle and tendon in adolescents and (b) to detect potential factors that could promote tendon overuse pathology. Therefore, the present study investigated the properties of the knee extensor muscle-tendon unit of male and female adolescent elite volleyball athletes compared to untrained boys and girls. With regard to the seemingly lower responsiveness of the tendon to plyometric loading compared to muscle (Kubo et al., 2007; Sáez-Sáez de Villarreal et al., 2010; Bohm et al., 2014) and our recent findings of a deficient modulation of tendon stiffness in relation to muscle strength development in adolescent athletes (Mersmann et al., 2016), we hypothesized to find markedly greater muscle strength in volleyball athletes compared with untrained adolescents, mediated by greater muscle thickness and fascicle pennation angles but only moderately higher tendon stiffness. The resultant higher mechanical demand (i.e., strain) placed upon the tendon during maximum effort muscle contractions could have important implications for the risk of tendon overload injury in adolescent volleyball athletes.

## Materials and methods

### Participants and experimental design

Twenty-four recreationally active adolescents [12 males, 12 females; ≤ 4 h of training per week, including school sports over the last 12 month (average values were 2.6 ± 0.9 h); henceforth referred to as *controls*] and 21 similar-aged elite volleyball athletes (12 male and 9 female athletes of the junior national team; ≥16 h of sport-specific training per week) participated in the present study. At the time of data acquisition the athletes had participated in national elite training for 9 ± 5 month, which comprised ~3 h of strength training, ~4 h of athletic training (i.e., jump and sprint drills, and core stability training) and ≥9 h of ball practice per week. As the effects of oral contraceptives (OC) on *in vivo* patellar tendon properties are most likely negligible (Hansen et al., 2013), we decided to include girls using OC in the present study (Athletes: *n* = 1/9; controls: *n* = 2/12; none of which were long-term OC users, i.e., <1 year of use).

The study was carried out in accordance with the recommendations of the university ethics committee with written informed consent from all subjects. All subjects (and the respective legal guardians when necessary) gave written informed consent in accordance with the Declaration of Helsinki. The protocol was approved by the university ethics committee. The measurements of muscle strength (i.e., knee extension moments), vastus lateralis architecture and patellar tendon mechanical properties were carried out on the dominant leg (i.e., leading leg in the spike jump in athletes or leg used for kicking a ball in controls) following a standardized warm-up including 5 min of ergometer cycling, 10 submaximal jumps and 10 submaximal isometric knee extension contractions as accustoming and preconditioning.

### Measurement of maximum knee joint moment

**Figure 1:**
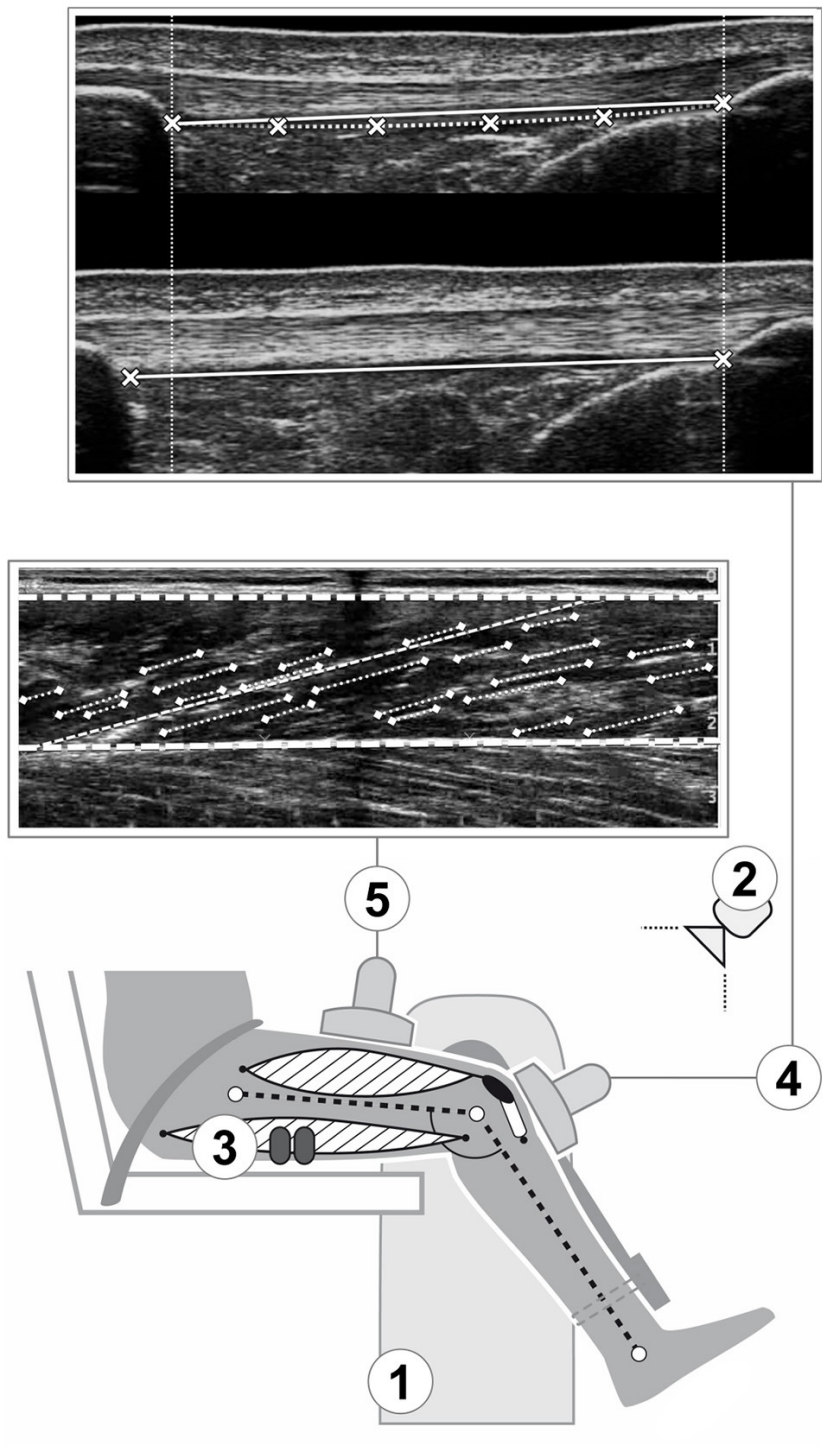
Schematic representation of the experimental setup. A dynamometer (1) was used to measure knee joint moments, while kinematic recordings (2) were used for inverse dynamics and electromyography (3) for the consideration of antagonist coactivation. Ultrasound imaging was integrated to assess patellar tendon elongation (4; the crosses indicate the reference points at the deep insertion sites and lower border of the tendon used for measuring elongation and rest length respectively; see methods section for details) and vastus lateralis architecture (5; the digitalization of the aponeuroses and the fascicle portions, indicated by the thick dashed line and pointed lines respectively, and the calculated reference fascicle, represented by the thin dashed line, are overlayed over the ultrasound image).

For the assessment of knee extensor muscle strength, the participants performed three maximum voluntary isometric knee extension contractions (MVC) on a dynamometer (Biodex Medical System 3, Shirley, NY, USA) at resting knee joint angles of 65°, 70°, and 75° (neutral full knee extension = 0°, values refer to the joint angle determined via the dynamometer). The resting angles were chosen based on our experience that participants reach their approximate optimum angle during contractions from these starting positions. The trunk angle was set to 85° (neutral full hip extension = 0°) and the hip as fixed to the dynamometer seat using a non-elastic strap (Figure 1). Kinematic data were recorded using a Vicon motion capture system (version 1.7.1; Vicon Motion Systems, Oxford, UK) integrating eight cameras operating at 250 Hz. Five reflective markers were fixed to the following anatomical landmarks: greater trochanter, lateral, and medial femoral epicondyles, and malleoli. The electromyographic (EMG) activity of the lateral head of the biceps femoris was recorded using two bipolar surface electrodes (Blue Sensor N, Ambu GmbH, Bad Nauheim, Germany) fixed over the mid-portion of the muscle belly with an inter-electrode distance of 2 cm after shaving and cleaning the skin. EMG data was captured at 1,000 Hz (Myon m320RX; Myon, Baar, Switzerland) and transmitted to the Vicon system via a 16-channel A-D converter.

Due to the non-rigidity of the human-dynamometer system (Arampatzis et al., 2004), the knee joint angle that was reached in the trails where the maximum knee extension moments were generated was 54 ± 4°, calculated in post-processing from the kinematic model. The resultant knee joint moments were calculated based on the inverse dynamics approach proposed by Arampatzis et al. (2004), which takes into account the inevitable axes misalignments of the knee joint and dynamometer during the course of the contraction as well as moments due to gravity. To account for the latter, an additional trial was recorded, where a passive knee extension was driven by the dynamometer at 5°/s with the shank of the participants fixed to the dynamometer lever pad. The contribution of the antagonistic muscles during the isometric contractions was estimated in the calculation of the maximum knee extension moments using the approach described by Mademli et al. (2004). In short, an EMG-activity knee flexion moment relationship was established on the basis of two additional knee flexion trials featuring an EMG-activity that was slightly lower and higher compared to the activity registered during the maximum knee extension trials, respectively.

### Measurement of vastus lateralis muscle architecture

Vastus lateralis architecture was assessed at a knee joint angle of 60°, which has been reported earlier to be the approximate optimum knee angle for force production of the knee extensors (Herzog et al., 1990). A 10-cm linear ultrasound probe (7.5 MHz; My Lab60; Esaote, Genova, Italy; probe: linear array (LA923), depth: 7.4 cm, focal point: 0.9 and 1.9, no image filter) was placed over the belly of the inactive muscle in its longitudinal axis at ~60% thigh length (average location of the maximum anatomical cross-sectional area; Mersmann et al., 2015). The ultrasound images were analyzed offline using a custom written MATLAB interface (version R2012a; MathWorks, Natick, MA, USA). The upper and deeper aponeuroses were defined by setting reference points along the aponeuroses that were approximated by a linear least squares fitting. Subsequently, we digitized the visible features of multiple fascicles (on average 22 ± 5) and calculated a reference fascicle based on the average inclination of the fascicle portions and the distance of the aponeuroses (Figure 1; Mersmann et al., 2014). The pennation angle refers to the angle between the reference fascicle and the deeper aponeurosis and fascicle length is reported normalized to femur length (average value of two measurements from the greater trochanter to the lateral epicondyle, identified by palpation, by means of a measuring tape).

### Measurement of patellar tendon mechanical properties

To establish the force-elongation behavior of the patellar tendon, the ultrasound probe was fixed overlying the patellar tendon in the sagittal plane using a modified knee brace (i.e., similar probe and settings as described above). The elongation of the tendon was captured at 25 Hz during five trials of isometric ramp contractions (i.e., steadily increasing effort from rest to maximum in ~5 s). The resting knee joint angle for the ramp contractions was chosen for each participant according to the MVC trial in which the highest moments were achieved.

The knee extension moments were calculated using the same considerations as described above for the MVC measurement (i.e., experimental setup, inverse dynamics approach, and correction for antagonistic contribution; see 2.2). The tendon force was then calculated by dividing the knee extension moment by the tendon moment arm. In 19 athletes it was possible to assess the moment arm directly from magnetic resonance images as described earlier (Mersmann et al., 2014). In all other participants, the moment arms were predicted based on sex, body height and mass, using the regression equation reported by Mersmann et al. (2016). The moment arms were adjusted to the respective knee joint angle position using the data of Herzog and Read (1993).

The ultrasound images were synchronized offline with the data recorded with the Vicon system using an externally induced voltage peak, which could be identified in both the ultrasound images and the analog data stream. Patellar tendon elongation during the contractions (Figure 1) was determined manually by tracking the deep insertion of the tendon at the patellar apex and the tibial tuberosity frame-by-frame using a custom-written MATLAB interface. To account for tendon slackness at rest, elongation was measured when the distance between the deep insertion points exceeded tendon rest length, which in turn was measured using a spline fit through the deep insertion marks and four additional points along the lower border of the slack tendon. The tracking was done by two experienced observers (F.M. and G.C.) and the force-elongation relationship of the five trials of each participant was averaged, using the highest common force value as peak force. This approach provides excellent reliability (≥0.95) and observer-independence (Schulze et al., 2012). The resultant force-elongation curve was fitted using a second-order polynomial, and tendon stiffness was calculated between 50 and 100% of the peak tendon force. As the length of a tendon has significant effects on its stiffness (Butler et al., 1978), we accounted for the anthropometric differences between the groups by also calculating *normalized tendon stiffness* (i.e., the product of stiffness and rest length). In one participant (male, control group), it was not possible to analyze patellar tendon elongation due to ultrasound image artifacts during the contraction.

### Statistics

The statistical analysis was conducted in SPSS (version 20.0; IBM, Armonk, NY, USA). We performed a two-way analysis of variances with the fixed factors training (i.e., controls, athletes) and sex (i.e., male, female). Normality of the standardized residuals was tested using the Kolmogorov–Smirnov test with Lilliefors correction (Lilliefors, 1967) and Levene's test was applied to test homoscedasticity. If normality or homoscedasticity was violated, we separately tested differences between athletes and controls, and males and females respectively, using the Mann-Whitney-Wilcoxon test and Bonferroni adjustment (adjusted *p*-values, denominated *p*_adj_, will be reported). The alpha level for all tests was set to 0.05. The effect size *f* for significant observations were calculated in G^*^Power (Version 3.1.6; HHU, Düsseldorf, Germany; Faul et al., 2007), based on either the partial eta squared or the group means and pooled standard deviation (for non-parametrically tested parameters). The subscript *Training* or *Sex* indicates if the effect size refers to differences between controls and athletes or males and females, respectively. Effect sizes of 0.1 ≤ *f* < 0.25 will be referred to as small, 0.25 ≤ *f* < 0.5 as medium and *f* ≥ 0.5 as large (Cohen, 2013).

## Results

There was no significant difference of age between the four groups (*p* = 0.9), but athletes compared to controls as well as males compared to females had significantly greater body height (*f*_Training_ = 1.91, *p* < 0.001; *f*_Sex_ = 1.27, *p* < 0.001), mass (*f*_Training_ = 0.50, *p*_adj_ = 0.004; *f*_Sex_ = 0.46, *p*_adj_ = 0.008) and femur lengths (*f*_Training_ = 1.60, *p* < 0.001; *f*_Sex_ = 0.56, *p* = 0.001; Table 1, respectively). There were no significant training-by-sex interactions on the anthropometric parameters (*p* > 0.05).

**Table 1:** Antrhopometric data of the adolescent controls and volleyball athletes.

	**Controls**		**Athletes**	
	**Male (*n* = 12)**	**Female (*n* = 12)**	**Male (*n* = 12)**	**Female (*n* = 9)**
Age [years]	16.8 ± 1.1	16.6 ± 0.9	16.8 ± 1.0	16.7 ± 0.9
Height [cm]^*^^#^	175.0 ± 4.4	162.1 ± 8.1	195.2 ± 4.0	181.6 ± 3.4
Mass [kg]^*^^#^	67.9 ± 11.5	61.9 ± 17.7	86.1 ± 7.2	68.0 ± 5.8
Femur length [cm]^*^^#^	41.5 ± 2.3	38.1 ± 3.6	48.4 ± 1.5	46.4 ± 1.9

*Values are means ± standard deviation*.

* *Significant difference between athletes and controls*,

# *significant difference between males and females, p < 0.05*.

Absolute (*f*_Training_ = 1.07, *p* < 0.001; *f*_Sex_ = 1.11, *p* < 0.001) and normalized knee extensor muscle strength (*f*_Training_ = 0.48, *p* = 0.004; *f*_Sex_ = 0.51, *p* = 0.002), and absolute (*f*_Training_ = 1.18, *p* < 0.001; *f*_Sex_ = 1.09, *p* < 0.001) and normalized resultant knee joint moments (*f*_Training_ = 0.56, *p* = 0.001; *f*_Sex_ = 0.45, *p* = 0.006) were significantly greater in athletes and males, respectively (Table 2), without significant training-by-sex interactions (*p* > 0.05). Further, there was a tendency toward lower coactivation in athletes (*f*_Training_ = 0.32, *p*_adj_ = 0.056), yet no significant differences between males and females (*p*_adj_ = 1.0; Table 2).

**Table 2:** Knee joint moments and antagonistic coactivation of adolescent controls and volleyball athletes.

	**Controls**		**Athletes**	
	**Male (*n* = 12)**	**Female (*n* = 12)**	**Male (*n* = 12)**	**Female (*n* = 9)**
MVC [Nm]^*^^#^	264.2 ± 57.2	192.8 ± 25.5	385.6 ± 48.3	260.6 ± 48.0
Normalized MVC [Nm/kg]^*^^#^	3.89 ± 0.52	3.25 ± 0.67	4.52 ± 0.74	3.86 ± 0.74
Resultant moment [Nm]^*^^#^	237.9 ± 54.2	174.7 ± 22.2	355.2 ± 38.7	245.2 ± 45.2
Normalized resultant moment [Nm/kg]^*^^#^	3.50 ± 0.54	2.96 ± 0.66	4.15 ± 0.58	3.63 ± 0.68
Antagonistic coactivation [%]^(*)^	13.0 ± 7.9	11.3 ± 6.4	9.1 ± 6.0	7.0 ± 3.4

*Values are means ± standard deviation. Moments were normalized to body mass. Antagonistic coactivation is the antagonistic moment normalized to the resultant knee joint moment. MVC, Maximum voluntary contraction*.

* *Significant difference between athletes and controls*,

# *Significant difference between males and females, p < 0.05*;

(*) *tendency toward a difference between controls and athletes, p_adj_ = 0.056*.

**Figure 2:**
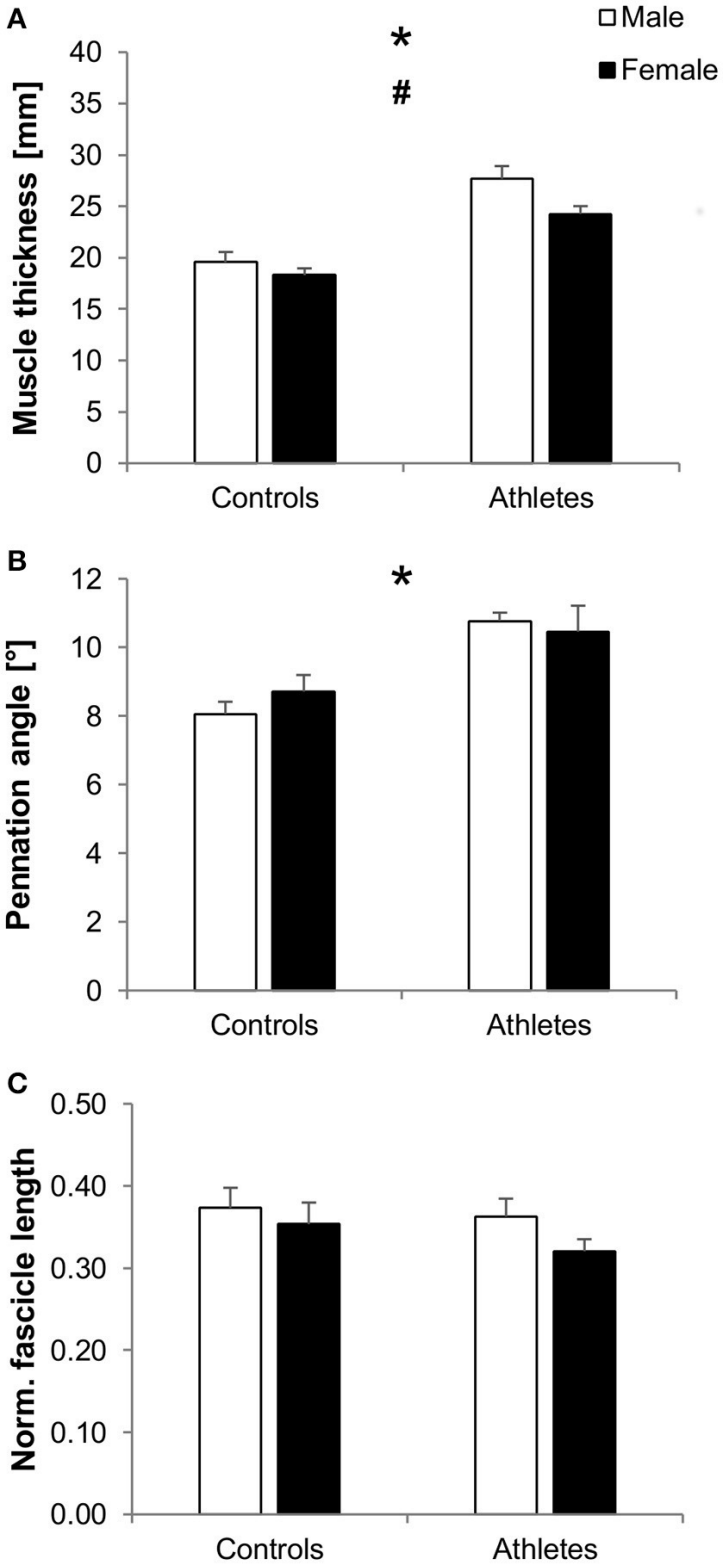
Mean values and standard error (error bars) of vastus lateralis muscle thickness **(A)**, pennation angle **(B)**, and normalized fascicle length (**C**; normalized to femur length) of male (white) and female (black) adolescent controls and volleyball athletes. ^*^Significant difference between athletes and controls, ^#^significant difference between males and females, *p* < 0.05.

We found significantly greater vastus lateralis muscle thickness in athletes compared to controls (*f*_Training_ = 1.12, *p* < 0.001) as well as in males compared to females (*f*_Sex_ = 0.37, *p* = 0.023), without significant training-by-sex interactions (*p* = 0.29; Figure 2A). Further, athletes featured greater pennation angles compared to controls (*f*_Training_ = 0.75, *p* < 0.001), while there were no significant differences between males and females (*p* = 0.72) or training-by-sex interactions (*p* = 0.3; Figure 2B). No significant effects or interactions were found on normalized fascicle length (*p* > 0.05; Figure 2C).

**Figure 3:**
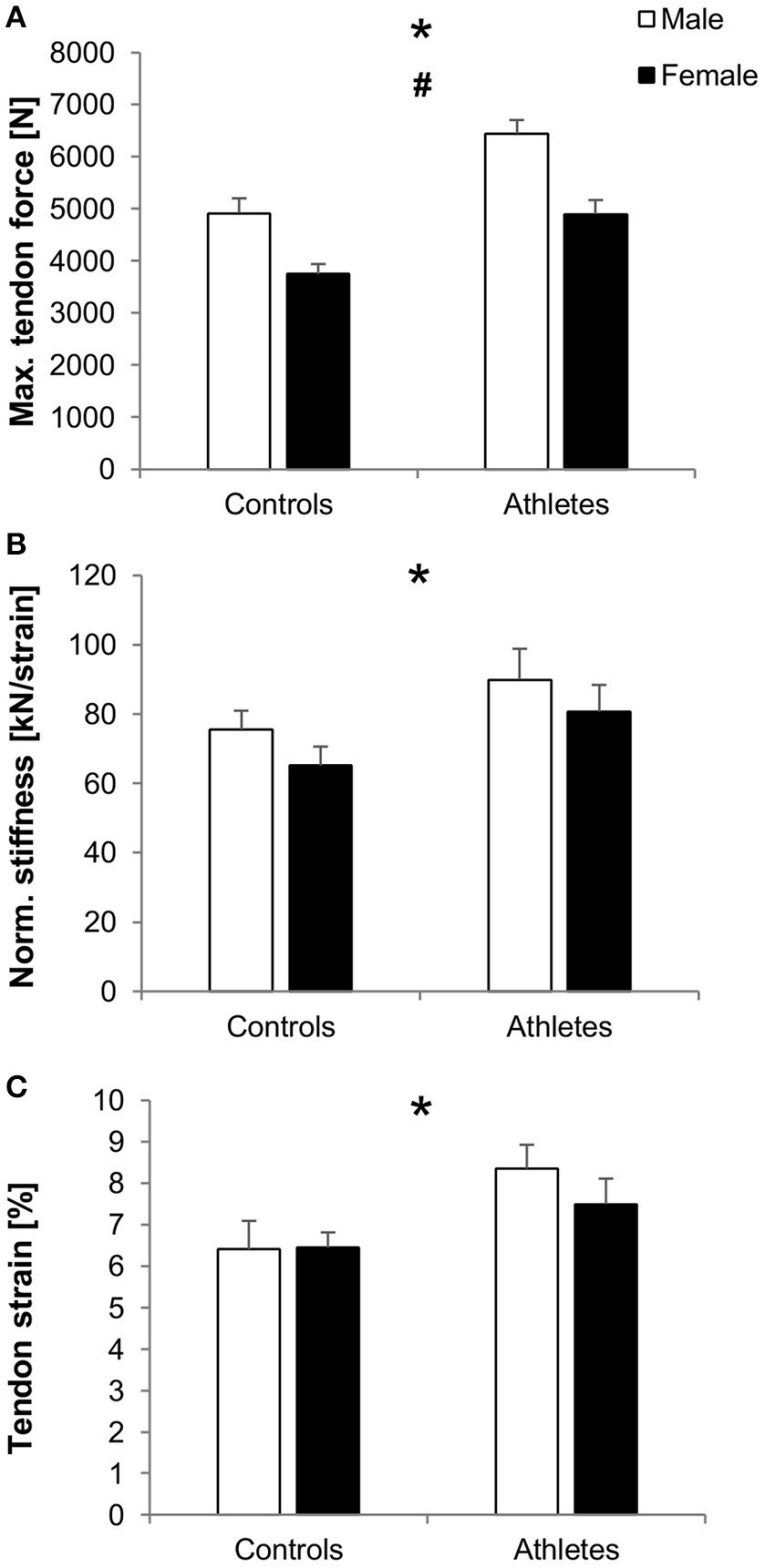
Mean values and standard error (error bars) of patellar tendon force **(A)**, normalized stiffness **(B)** and strain during maximum isometric contractions **(C)** of male (white) and female (black) adolescent controls and volleyball athletes. ^*^Significant difference between athletes and controls, ^#^significant difference between males and females, *p* < 0.05.

In athletes compared to controls, we found greater maximum patellar tendon force (*f*_Training_ = 0.82, *p* < 0.001; Figure 3A), normalized tendon stiffness (*f*_Training_ = 0.34, *p* = 0.04; Figure 3B), tendon strain (*f*_Training_ = 0.42, *p*_adj_ = 0.016; Figure 3C) and elongation during maximum contractions (*f*_Training_ = 0.55, *p* = 0.001; Table 3), tendon moment arm (*f*_Training_ = 1.58, *p* < 0.001; Table 3), and rest length (*f*_Training_ = 0.57, *p* = 0.001; Table 3), but no significant difference in absolute tendon stiffness (*p* = 0.26; Table 3). Tendon force (*f*_Sex_ = 0.83, *p* < 0.001), moment arm (*f*_Sex_ = 1.55, *p* < 0.001) and rest length (*f*_Sex_ = 0.48, *p* = 0.004) were higher in males compared to females. However, there were no significant differences between sexes with regard to absolute (*p* = 0.57) or normalized tendon stiffness (*p* = 0.17), strain (*p* = 0.49) and elongation (*p* = 0.12) or training-by-sex interactions in general (*p* > 0.05).

**Table 3:** Patellar tendon properties of adolescent controls and volleyball athletes.

	**Controls**		**Athletes**	
	**Male (*n* = 11)**	**Female (*n* = 12)**	**Male (*n* = 12)**	**Female (*n* = 9)**
Moment arm [mm]^*^^#^	53.6 ± 1.3	49.2 ± 2.0	59.8 ± 3.2	53.5 ± 3.0
Rest length [mm]^*^^#^	51.6 ± 4.5	46.6 ± 4.5	56.0 ± 5.8	52.5 ± 3.4
Stiffness [N/mm]	1474 ± 375	1392 ± 352	1650 ± 670	1560 ± 518
Maximum elongation [mm]^*^	3.30 ± 1.26	2.98 ± 0.64	4.71 ± 1.37	3.94 ± 1.05

*Values are means ± standard deviation*.

* *Significant difference between athletes and controls*,

# *Significant difference between males and females, p < 0.05*.

## Discussion

The present study investigated the effects of mechanical loading in terms of athletic volleyball training on the properties of the knee extensor muscle-tendon unit in male and female adolescents. The results demonstrate that, irrespective of sex and anthropometric differences, both muscle and tendon show clear differences between trained and untrained boys and girls. We found a significantly greater muscle strength capacity in the athletes compared to the control group and corresponding differences in vastus lateralis muscle thickness and pennation angle. Similarly, athletes demonstrated greater normalized patellar tendon stiffness, indicating that the tendon adapts to mechanical loading before full maturation of the musculoskeletal system. However, the only medium effects of training status on normalized tendon stiffness compared to the large effects on tendon force (*f* = 0.34 and 0.84, respectively) suggest an imbalance in the adaptation of muscle and tendon, subjecting the tendons of athletes to higher levels of strain during maximum contractions compared to controls. Therefore, our hypotheses were confirmed.

It is well established that physical training increases muscle strength even in childhood (Falk and Eliakim, 2003; Matos and Winsley, 2007; Legerlotz et al., 2016) and, thus, our findings of greater knee extensor strength in trained compared to untrained adolescents were not surprising. Similarly, our findings of greater vastus lateralis thickness, pennation angle, and, in tendency, reduced antagonistic coactivation in athletes are in line with earlier reports of muscle morphological (Fukunaga et al., 1992; Kanehisa et al., 1995a, 2003; Daly et al., 2004; Mersmann et al., 2016) and neuronal adaptations (Ramsay et al., 1990; Ozmun et al., 1994) as potential contributors to training-induced increases of strength in young athletes. However, this is, to our knowledge, the first study to report differences in patellar tendon mechanical properties between an athletic and untrained adolescent population. Waugh et al. (2014) investigated the effects of a strength training intervention on the mechanical properties of the Achilles tendon in pre-pubertal children and reported a significant increase of tendon stiffness. More recently, a cross-sectional comparison of tendon thickness between 500 adolescent athletes from different sports provided an indication of tendon plasticity in response to increased loading for the patellar tendon as well (Cassel et al., 2016). Earlier work of our group already suggested that the patellar tendon is responsive to mechanical stimulation, but it was not possible to differentiate the effects of mechanical loading and maturation (Mersmann et al., 2017) or to directly compare the mechanical properties of the patellar tendon between athletes and controls due to inhomogeneous sample composition with regard to sex (Mersmann et al., 2016). The different normalized patellar tendon stiffness between athletes and controls in the present study provide evidence that also the patellar tendon adapts to mechanical loading before adulthood. With regard to the marked effect of athletic training on muscle strength, the increase of tendon stiffness serves two important mechanical functions. First, it improves the performance capacity of the muscle-tendon unit. Higher tendon stiffness is associated with a reduction of electromechanical delay (Waugh et al., 2014), increased rate of torque development (Bojsen-Møller et al., 2005; Waugh et al., 2013) and jump performance (Bojsen-Møller et al., 2005; Burgess et al., 2007), and it maintains muscle fascicle kinetics within an optimal operating range when the force production during a movement task (e.g., jumping) increases due to training (Lichtwark and Wilson, 2007). Second, the modulation of stiffness may serve as a protective mechanism against unphysiological levels of strain induced by the increased force-generating capacity of the trained muscle, since the ultimate strain of tendons is considered to be relatively constant (Abrahams, 1967; Loitz et al., 1989; LaCroix et al., 2013; Shepherd and Screen, 2013). However, besides increased normalized stiffness, the athletes in the present study featured increased levels of tendon strain during maximum muscle contractions as well, which is in accordance with earlier findings of our group (Mersmann et al., 2016) and indicates an imbalanced adaptation of muscle and tendon. Tendon strain during maximum contractions is an indicator of how the integrity of the tissue is challenged by the working muscle. Wren et al. (2003) demonstrated that the initial strain induced in the tendon by a given load predicts the lifetime of the tendinous tissue during static and cyclic loading. The increased demand placed upon the tendon by the working muscle we observed in athletes could increase the risk of accumulating microdamage and predispose for tendon injury (Butler et al., 1978; Fung et al., 2009; Legerlotz et al., 2013), especially in athletes that are subjected to high frequencies of maximum jumping in their sportive activity (Bahr and Bahr, 2014). The high prevalence of tendinopathy in volleyball athletes (Lian et al., 2005) and *in vivo* reports of increased levels of tendon strain in patients with tendinopathy (Arya and Kulig, 2010; Child et al., 2010) further support the idea that an imbalanced adaptation of muscle and tendon in athletes might increase the risk to develop tendinopathy, though certainly more direct evidence is needed to support this assumption.

The higher normalized stiffness compared to untrained adolescents as well as the imbalance between muscle strength and tendon stiffness are most likely related to the type of mechanical loading the athletes are subjected to. Systematic investigations on the specific effects of different modes of mechanical stimulation on human tendons *in vivo* demonstrated that high magnitude loading (in terms of tendon force and corresponding strain) effectively promote tendon stiffness (Arampatzis et al., 2007, 2010; Kongsgaard et al., 2007; Malliaras et al., 2013). High-level strain application has been associated with greater tendon cell deformation (Arnoczky et al., 2002) and collagen fiber recruitment (Kastelic et al., 1980; Hansen et al., 2002), which are considered important factors for the transmission of extracellular matrix strains into cellular responses (Lavagnino et al., 2008). Direct measurements (Finni et al., 2000) and estimations of patellar tendon forces (Janssen et al., 2013) suggest tendon loads to exceed five times body weight during jumping and landing, which makes it reasonable to assume that athletic volleyball training provides sufficient loading in terms of load magnitude to induce tendon adaptation. High tendon strain rates during plyometric loading and the associated fluid-flow dependent shear stress on tendon cells might additionally stimulate tendon metabolism (Haut and Haut, 1997; Archambault et al., 2002; Lavagnino et al., 2008). Interestingly though, most longitudinal intervention-studies that applied plyometric exercise (over 8–14 weeks) failed to elicit significant adaptive changes of human tendons (Kubo et al., 2007; Fouré et al., 2009, 2010; Houghton et al., 2013), even at high loading magnitudes (Bohm et al., 2014). In consequence, an increase of muscle strength without an adequate modulation of stiffness induced by plyometric loading can result in increased levels of tendon deformation (Kubo et al., 2007), which is commonly not observed following high-intensity loading with long contraction durations (e.g., Kongsgaard et al., 2007; Arampatzis et al., 2007, 2010; Malliaras et al., 2013). It has been argued that short strain durations of plyometric regimen or high-frequency load-relaxation cycles might compromise the effectiveness of the mechanotransduction at the tendon level (Arampatzis et al., 2010; Bohm et al., 2014). Therefore, it seems well possible that only long-term habitual plyometric loading results in a notable modulation of tendon stiffness, but that the effects are still less pronounced compared to the associated and more clearly established increases of muscle strength (Sáez-Sáez de Villarreal et al., 2010). With regard to the greater body mass of the athletes in our study, it cannot be excluded, of course, that increased habitual loading during everyday activities partly contributed to the greater tendon stiffness compared to the control group. However, body mass was not correlated to normalized tendon stiffness in the present study (*r* = 0.013, *p* = 0.94) and data by Waugh et al. (2011) sugests that, at least in the Achilles tendon, a clear association between body mass and tendon stiffness only exists during pre-pubertal growth and not in adulthood. Thus, it is reasonable to conclude that the sport-specific loading was the main determinant for both the greater normalized tendon stiffness as well as the imbalance between muscle strength and tendon mechanical properties.

An alternative or additional explanation for the imbalance between muscle strength and tendon stiffness, indicated by the increased levels of tendon strain during maximum contractions in athletes compared to controls, could be differences in the plasticity of muscle and tendon as a function of maturation. Earlier work of our group already provided evidence that under the influence of maturation and superimposed mechanical loading the development of muscle morphology and function might precede adaptive and developmental processes at the tendon level in adolescent volleyball athletes (Mersmann et al., 2014, 2017). The results of a recent meta-analysis indicate an increase in the responsiveness of the neuromuscular system to mechanical loading early in adolescence (Moran et al., 2016), which is likely in part related to the muscle-anabolic effects of sex hormones (Vingren et al., 2010; Hansen and Kjaer, 2014). The effects of the rapid increase of circulating sex hormones on tendon plasticity during growth on the other hand are basically unknown (Hansen and Kjaer, 2014). Evidence on human adults suggest that estrogens reduce collagen synthesis in response to exercise, indicating a reduced adaptability of the tendinous system in women compared to men (Hansen and Kjaer, 2014; for reviews see Magnusson et al., 2007). In the present study, however, the differences between athletic and untrained adolescents of both patellar tendon mechanical properties and tendon strain (as an indicator of musculotendious imbalances) were similar in boys and girls (i.e., no significant training-by-sex interaction: *p* = 0.94 and 0.44, respectively). Therefore, it is well possible that sex-related differences in the responsiveness of tendinous tissue to mechanical loading unfold with the formation of the tendon core tissue at the end of adolescence (Heinemeier et al., 2013) and long-term exposure to elevated levels of circulating estrogens (Bryant et al., 2008; Hansen and Kjaer, 2014). However, due to a lack of comparable data on adult volleyball athletes with a similar training history compared to the adolescents of the present study, the additional influence of maturation on the balance of musculotendinous adaptations to loading still remains an assumption. Clinical evidence shows that the probability of non-contact soft-tissue injury in adults rises when training loads are increased rapidly (Gabbett, 2016) and differences between muscle and tendon in the responsiveness to distinct mechanical stimuli (Arampatzis et al., 2007, 2010; Kubo et al., 2007) as well as in the time course of adaptation (Kubo et al., 2010, 2011a) are issues that affect the balance of muscle and tendon adaptation in adult athletes as well. Nevertheless, it is evident that there is a need to increase our understanding of the complex interaction of mechanical loading and changes of the hormonal milieu on tendon plasticity in general, and with regard to adolescence in particular.

The World Health Organization defines adolescence as a period ranging roughly from 10 to 19 years of age (World health organization: department of child adolescent health development, 2001), yet a distinction between early adolescence (i.e., 10–14 years) and late adolescence (i.e., 15–19 years) has become common more recently (Sawyer et al., 2012) to partly account for the drastic physical, cognitive and socio-emotional development during the overall period of adolescence. Since it is barely possible to adequately control for biological age with non-invasive measures (Malina et al., 2015), this study investigated late adolescent cohorts following the assumption that the differences in biological and chronological age are most pronounced in early adolescence. Therefore, it is unclear if the observations of the present study are representative for other stages of development. Considering the observations of increased tendon stress during the longitudinal growth of the muscle-tendon unit in early adolescence (Neugebauer and Hawkins, 2012), this could be important maturational phase to address with future experimental designs.

Due to the cross-sectional design of the study, the time course of muscle and tendon adaptation in relation to training history (i.e., 6 ± 3 years of volleyball practice, 9 ± 5 month of elite-level training) and/or maturation also remains unclear. The fluctuations of both muscle and tendon properties during a competitive season, which was observed in a recent study of our group (Mersmann et al., 2016), suggests that also the time point of data acquisition could be important for a comparison to untrained individuals. In the present study, it was not possible to adjust the scheduling of the measurements to a specific time point in the competitive season of the athletes. However, since the fluctuations over time reported earlier (e.g., MVC: 5%, stiffness: 8%, strain: 14%; Mersmann et al., 2016) were markedly lower as the differences observed between the athletes and controls of the present study (MVC: 45%, normalized stiffness: 23%, strain: 24%) it is unlikely that this limitation affected our main findings and conclusions.

The selection of elite volleyball athletes as trained group was based on the sport-specific loading profile (i.e., high intensity plyometric loading) and the urgent need for a better understanding of muscle and tendon adaptation due to the high incidence of tendinopathy in that group (Lian et al., 2005). The generalizability of our findings to other athletic populations is speculative. It seems well possible that differentially graded adaptations of muscle and tendon might occur in other sports that incorporate types of loading that lead to rapid strength gains (i.e., high intensity muscle contractions; Seynnes et al., 2007; DeFreitas et al., 2011) or more effectively stimulate muscle compared to tendon adaptation (i.e., moderate intensity loading, plyometric loading; Arampatzis et al., 2007, 2010; Kubo et al., 2007; Bohm et al., 2014). Our findings might therefore be relevant for the design of training programs in sports such as Basketball, Soccer or athletic jumping disciplines as well. However, this assumption warrants verification in future studies.

In conclusion, the present study provides evidence that, irrespective of sex, adolescent volleyball athletes feature markedly greater muscle strength, mediated by greater muscle thickness and pennation angle as well as reduced antagonistic coactivation, and greater normalized tendon stiffness compared to untrained adolescents. However, increased levels of tendon strain during maximum contractions in athletes indicate an imbalance in the development of muscle strength and tendon stiffness that might be partly due to (a) suboptimal tendon mechanical stimulation by sport-specific loading and (b) deviations in the temporal dynamics of muscle and tendon adaptation during adolescence. The potential contribution of musculotendinous imbalances to the increasing risk of tendon overload injury during adolescence (Stracciolini et al., 2014; Simpson et al., 2016) highlight the importance to further increase our understanding of muscle and tendon plasticity during growth and maturation as well as to evaluate the potential of implementing loading regimen that effectively facilitate tendon mechanical properties (Bohm et al., 2015) into the athletic training of adolescents for injury prevention.

## Author contributions

FM and AA conceived the experiment; FM, GC, and SB performed the experiments; all authors substantially contributed to data analysis; FM and AA interpreted the data; FM drafted the manuscript and GC, SB, and AA made important intellectual contributions during revision. All authors approved the final version of the manuscript and agree to be accountable for the content of the work.

### Conflict of interest statement

The authors declare that the research was conducted in the absence of any commercial or financial relationships that could be construed as a potential conflict of interest. The reviewer CC and handling Editor declared their shared affiliation, and the handling Editor states that the process nevertheless met the standards of a fair and objective review.
